# Chronic inflammatory systemic diseases: An evolutionary trade-off between acutely beneficial but chronically harmful programs

**DOI:** 10.1093/emph/eow001

**Published:** 2016-01-27

**Authors:** Rainer H. Straub, Carsten Schradin

**Affiliations:** ^1^Laboratory of Experimental Rheumatology and Neuroendocrine Immunology, Division of Rheumatology, Department of Internal Medicine, University Hospital Regensburg, Regensburg, Germany;; ^2^Université De Strasbourg, IPHC-DEPE, 23 Rue Becquerel, Strasbourg 67087, France;; ^3^CNRS (Centre National De La Recherche Scientifique), UMR7178, Strasbourg 67087, France;; ^4^School of Animal, Plant and Environmental Sciences, University of the Witwatersrand, Johannesburg, South Africa

**Keywords:** inflammatory systemic disease, neuroendocrine immunology, energy regulation, disease sequelae

## Abstract

It has been recognized that during chronic inflammatory systemic diseases (CIDs) maladaptations of the immune, nervous, endocrine and reproductive system occur. Maladaptation leads to disease sequelae in CIDs. The ultimate reason of disease sequelae in CIDs remained unclear because clinicians do not consider bodily energy trade-offs and evolutionary medicine. We review the evolution of physiological supersystems, fitness consequences of genes involved in CIDs during different life-history stages, environmental factors of CIDs, energy trade-offs during inflammatory episodes and the non-specificity of CIDs. Incorporating bodily energy regulation into evolutionary medicine builds a framework to better understand pathophysiology of CIDs by considering that genes and networks used are positively selected if they serve acute, highly energy-consuming inflammation. It is predicted that genes that protect energy stores are positively selected (as immune memory). This could explain why energy-demanding inflammatory episodes like infectious diseases must be terminated within 3–8 weeks to be adaptive, and otherwise become maladaptive. Considering energy regulation as an evolved adaptive trait explains why many known sequelae of different CIDs must be uniform. These are, e.g. sickness behavior/fatigue/depressive symptoms, sleep disturbance, anorexia, malnutrition, muscle wasting—cachexia, cachectic obesity, insulin resistance with hyperinsulinemia, dyslipidemia, alterations of steroid hormone axes, disturbances of the hypothalamic-pituitary-gonadal (HPG) axis, hypertension, bone loss and hypercoagulability. Considering evolved energy trade-offs helps us to understand how an energy imbalance can lead to the disease sequelae of CIDs. In the future, clinicians must translate this knowledge into early diagnosis and symptomatic treatment in CIDs.

## INTRODUCTION

Chronic inflammatory systemic diseases (CIDs) like rheumatoid arthritis, systemic lupus erythematosus, multiple sclerosis and many others are a burden to humans because of life-long debilitating illness, increased mortality and high costs for therapy and care. Other than CIDs, infectious disease like influenza or scarlet fever typically last only for a short period of time and they normally do not lead to chronic disease sequelae. The main difference between CIDs and acute infectious disease is span of time. While an acute infectious disease or inflammation during wound healing represents an adaptive response to overcome a disease and, thus, to increase life-time reproductive success, a CID is outside the adaptive reaction norm leading to maladaptive responses and a reduction of evolutionary fitness, because the stimulating trigger cannot be removed (for discussion of reaction norm see [1]).

CIDs are often discussed within the evolutionary medicine framework of the ‘hygiene hypothesis’ [2]. This model says that since the start of urbanization humans experienced a depletion of typical environmental infectious organisms such as helminths with which mammals co-evolved. The loss of these infectious agents provoked a change of normal background levels of immunoregulation during infancy, and this stimulated a more aggressive immune response in adulthood and old age and, thus, more frequent appearance of CIDs [2].

An approach of evolutionary medicine to CIDs is needed because of the fact that CIDs exist although they exert a negative effect on reproductive fitness [3–9]. So far, the persistence of CIDs was partly explained by the important roles a limited number of networked immune system genes play in pathogen defense and other functions that are under strong natural selection [10]. While these previous theories focused on the trigger of CIDs, here we extend this by focusing on common disease pathways and sequelae of CIDs.

Coordinated reactions of the supersystems (immune, nervous, endocrine and reproductive) that maintain homeostasis have been evolutionarily conserved to respond to and eliminate foreign agents over a period of days to a few weeks (e.g. influenza or scarlet fever) [11]. If the responses of these supersystems fail to return to normal such as in autoimmunity or other forms of systemic chronic inflammation (e.g. autoinflammatory syndromes), maladaptation of the supersystems perpetuates long-lasting CIDs [11, 12]. Since 2003 this theory has been refined, because new theoretical concepts from the field of bodily energy regulation were added [13–15]. This addition improved the model that is presented here in an updated version.

## EVOLUTION OF THE SUPERSYSTEMS AND THEIR INTERACTIONS

Infectious and non-infectious hazards were present throughout the entire evolutionary history of all vertebrates including humans. With rats and mice, we share many similar inflammatory mechanisms, although the last common ancestor lived 65 million years ago [16]. Humans and chicken use immunoglobulin gene rearrangement and somatic hypermutation to shape the antibody response towards infectious agents [16]. Even sharks have a very similar immune system like ours with T cell receptors and immunoglobulin diversity based on V(D)J recombination and the RAG1/2 system [17]. Differences only become apparent when comparing humans with jawless fish (lampreys and hagfish; the last common ancestor with humans lived 460 million years ago), which use a more primitive immune system which, nevertheless, already consists of variable lymphocyte receptors [17].

Thus, when we study the activation of the immune system in the context of the most prevalent danger of infection, we have to consider that the evolutionary history of the human immune system spans more than 420 million years. The immunological pathways are conserved and are very similar between sharks, birds, rodents and humans. In sum, acute inflammation is an adaptive response present in nearly all vertebrates, which has evolved to cope with infections or short-lived inflammatory responses (e.g. wound healing or foreign body reactions).

In a similar way, fish, birds, rodents and humans share many neuroendocrine pathways responsible for bodily homeostasis such as the hypothalamic-pituitary-adrenal axis (HPA) axis or the sympathetic nervous system. For example, glucocorticoids of the HPA axis are present in mammals, birds, jawed fish and even in lamprey [18, 19].

The bilaterally organized sympathetic nervous system is present in jawed fish like sharks and rays and in all vertebrates, but it is absent in lampreys and hagfish [20]. Nevertheless, lampreys produce catecholamines in monoamine-containing neurons, which also happens in leukocytes in the human body [21, 22]. Even bacteria possess functional orthologs of tyrosine hydroxylase, which converts L-thyroxine to L-DOPA, the first catecholamine in the synthesis cascade towards noradrenaline and adrenaline and bacteria use catecholamines as growth factors [23, 24].

In addition, central nervous system responses such as sickness behavior [25] or anorexia [26] are similar in birds and rodents, indicating long evolutionary history. Rainbow trouts can become anorectic under stressful conditions [27]. Even crocodiles develop anorexia and lethargy during infection [28], which demonstrates the uniformity of the response. In sum, the immune, endocrine, and nervous systems have evolved over hundreds of millions of years, and to understand diseases in humans, including CIDs, the long evolutionary history of these supersystems has to be taken into account.

When we observe crosstalk between the supersystems, many similar pathways are used in humans and other vertebrates to maintain homeostasis. If homeostasis is disturbed by stressful events such as infection (leading to inflammation), the different supersystems need to cooperate in order to overcome the threat. The crosstalk of supersystems has co-evolved with infectious agents. Importantly, such a reaction to inflammatory stress is relatively uniform in many species. Genes enabling a coordinated response of the supersystems, allowing the organism to overcome infection or wounding, will increase survival probability and the potential for future reproduction and—as such—evolutionary fitness.

## EVOLUTIONARY FITNESS AND CIDs

It can be expected that CIDs lead to reduced evolutionary fitness by indirect and direct effects. First, affected individuals would have reduced competitive abilities, reducing their access to resources such as food and sexual partners, and their social status in the group might be impaired. There might be even active repudiation in the group. In our ancestors, the risk of injury or even death in any competition including fights and warfare as well as encounters with predators would have significantly increased due to their handicap.

Second, CIDs directly influence reproductive success by decreasing fecundity [3–9]. Even with optimum treatment in our days, fertility is reduced in both men and women with CIDs [29]. CIDs are accompanied by a severe decrease of androgen levels [30–32], which can be observed even after subcutaneous injection of the proinflammatory cytokine interleukin-6 (IL-6) into healthy volunteers. IL-6 blocks the release of testosterone by ∼50%, and it needs 7 days until restoration of normal serum levels [33]. In the acute phase of inflammatory diseases, IL-6 is nearly always increased and, thus, affects sexual function and sex hormone release. During a CID, this cytokine remains elevated if no appropriate treatment is applied.

There is other clear evidence that the proinflammatory TNF directly inhibits gonadal cells responsible for testosterone secretion [34]. Injection of another inflammatory cytokine, IL-1β, into the brain of experimental animals decreased testosterone production for a long time [35]. The same cytokines influence sperm motility resulting in reduced mucosal penetration properties and decreased male fertility [36]. Similarly, female reproduction is influenced by inflammatory cytokines such as IL-1 and TNF [37, 38], and infectious episodes reduce female fertility [39]. Inflammation has deleterious effects on the menstrual cycle, leading to amenorrhea [40]. Thus, the reproduction system is switched-off in both sexes during inflammation.

From a point of view of evolutionary history, it can be expected that the disease itself lead to early death in our ancestors, because no appropriate therapies were available until the 1950s. All conditions imply a *high negative selection pressure* for specific genetic factors leading to CIDs, especially, if these would have occurred in young reproducing adults.

## GENETICS AND ENVIRONMENTAL INFLUENCE IN CIDs

### Accumulation theory of CIDs

Natural selection cannot favor any chronic disease, because of the associated fitness costs and absence of any fitness gains. However, an individual that would suffer from a CID at a post-reproductive life history stage might have transmitted genes—increasing the risk to develop a chronic illness in late age—during an earlier life history stage when it was healthy and reproducing.

Several genes that play a role in CIDs have been identified. The best know association between CIDs and genes has been established for the HLA system, which is an important element of antigen presentation. Sometimes the risk to develop a disease in presence of a certain subtype of the HLA system can be high. Approximately 10–25% of humans carry risk HLA genotypes but the prevalence of any of the CIDs is 0.1–1% [41], and thus, far below the expected 10–25%. Thus, there have to be additional factors and a certain HLA protein alone is not necessary and not sufficient to elicit a certain CID.

Today we label such an alteration as a genetic disease-promoting risk factor. As the alteration of an HLA protein is not the single causative factor, we expect that other genetic factors and the environment (see below) are also relevant. Indeed, CIDs have a multifactorial genetic background as demonstrated, e.g. in rheumatoid arthritis [42]. Many additional genetic factors have been described that increase the risk to develop rheumatoid arthritis (Table 1). Therefore, negative selection for specific HLA gene variants most probably was small, because in most individuals carrying the gene the disease did not develop due to the lack of additional factors. In contrast, polymorphic HLA genes have been retained because they helped to overcome various infections [43], leading to positive selection. The genetic prerequisites for a CID can be retained over generations and generations via individuals that never express the disease because relevant co-factors may either not occur or are expressed following very irregular patterns.

**Table 1.** Risk factors of rheumatoid arthritis

HLA system (HLA DR4 [DRB1*04])
tumor necrosis factor alpha-induced protein 3 (TNFAIP3) (negative regulator of the NF-kappaB pathway)
protein tyrosine phosphatase receptor type C (CD45) (receptor and protein tyrosine phosphatase)
interferon gamma receptor 2 (cytokine receptor)
CD40 (cell surface molecule and receptor)
tyrosine kinase 2 (TYK2) (signaling of cytokine receptors)
IL-6 receptor (cytokine receptor)
protein tyrosine phosphatase type 22 (PTPN22) (signaling of receptors)
Fc-gamma receptor II 2B (CD32, Fc fragment of IgG, low affinity IIb receptor)
IL-2 (cytokine)
IL-2 receptor alpha chain (cytokine receptor)
SH2B3 (member of the SH2B adaptor family of proteins, signaling of growth factor and cytokine receptors)
ICOSLG (inducible T-cell co-stimulator ligand)

The respective single nucleotide polymorphism in the mentioned genes have been found in genome-wide association studies [42]. The list is ordered according to the significance level of the statistical test for relative risk. HLA, human leukocyte antigen; IL, interleukin; TNF, tumor necrosis factor.

Table 1 demonstrates that the same signaling factors can play a role in different CIDs such as multiple sclerosis (*TYK2*, *CD40*, *TNFAIP3*, *PTPN22*, *IL-2*, *IL-2RA* and others; ref. [44]), type 1 diabetes mellitus (*TNFAIP3*, *PTPN22*, *IL-2*, *IL-2RA*, *SH2B3* and others, refs. [45–47]), systemic lupus erythematosus (*TYK2*, *TNFAIP3*, *PTPN22*, *FCGR2B*, reviewed in ref. [48]), and Crohn’s disease (*TYK2*, *PTPN22*, *IL-2RA*, *ICOSLG* and others, ref. [49]). This tells us that the same immunostimulatory or immunoinhibiting signaling pathways are used in the different diseases. These shared immune factors have been positively selected in the context of infectious diseases but not for CIDs (see next section ‘*Pleiotropy theory of CIDs*’, ref. [10]).

In addition, accumulation of different risk alleles does not only lead to additivity of risk but to synergistic effects as demonstrate for the R620W PTPN22 allele and ‘HLA-DRB1 shared epitope’ alleles in rheumatoid arthritis [50]. In addition, African Americans positive for ‘HLA-DRB1 shared epitope’ alleles have a higher risk of developing rheumatoid arthritis when they demonstrate a higher degree of European ancestry, which demonstrates genetic admixture [51].

From monozygotic twin studies, it is known that genes contribute to the appearance of CIDs (Table 2). In these twin studies, the concordance rate has been determined as the quantitative statistical expression for the concordance of a given genetic trait. The concordance rate varies between 0 and 50%, and is on average 25% for the mentioned CIDs in Table 2, indicating that the environment explains ∼75% of the variation. Thus, considering gene-environment interactions is of outstanding importance. The accumulation theory describes how the accumulation of several genetic and environmental risk factors increases the risk to develop a CID.

**Table 2.** Concordance rate in twin studies in chronic inflammatory systemic diseases

Disease	Monozygotic concordance rate (%)	Refs
Rheumatoid arthritis	0–21	[41, 52, 53]
Hashimoto thyroiditis	17	[54]
Systemic lupus erythematosus	11–24	[41, 55]
Multiple sclerosis	6–31	[41]
Graves’ disease	22	[41]
Type 1 diabetes mellitus	13–38	[41]
Psoriasis	35	[56]
Ankylosing spondylitis	50	[57]

An interesting example for an environmental factor affecting the risk to develop CIDs is smoking. Already in the 1970s, a link was demonstrated between smoking and increased serum levels of autoantibodies [58]. The first epidemiological studies on the link between smoking and the risk to develop rheumatoid arthritis appeared in the early 1990s [59–61]. One study on monozygotic twins showed that smoking increased the risk to develop rheumatoid arthritis by a factor of 12 [62]. The risk remains elevated for several years after smoking cessation [63]. This demonstrates that smoking is a very strong environmental risk factor, and Lars Klareskog et al. showed that smoking may trigger HLA-DR-restricted immune reactions to autoantigens modified by citrullination [64]. Importantly, smoking is also linked to an increased risk to develop other CIDs such as ankylosing spondylitis [65], multiple sclerosis [66], Crohn’s disease [67] and systemic lupus erythematosus [68, 69]. However, smoking can also reduce the risk of developing CIDs such as type 1 diabetes mellitus [70], ulcerative colitis [67] or pemphigus [71].

There are several other environmental factors that are known to affect the development pf CIDs. For example, alcohol can protect individuals from developing rheumatoid arthritis [72]. Intake of oily fish was associated with a modestly decreased risk of developing rheumatoid arthritis [73]. Another environmental risk factor for rheumatoid arthritis and other autoimmune diseases is silica exposure, which occurs in construction work (cement, demolition) [74, 75]. It was discussed that silica is a stimulator of the immune system, which may trigger the autoimmune process leading to chronic inflammation [74, 75]. These examples clearly demonstrate that environmental factors play an important role, supporting the accumulation theory.

Another environmental modulator is the microbiota in the gut and elsewhere on body surfaces. Over millennia of co-evolution, microbiota has been a strong stimulus for shaping the innate and adaptive immune system in vertebrates [76]. During evolution, responses towards microbes have established a fine-tuning of immune responses [43]. We expect that microbiota-reactive T and B lymphocytes support homeostasis of the body [76]. Thus, normal microbiota or changes in microbiota during disease states or continuous illness can modulate the expression of CIDs. The famous example of HLA-B27 transgenic rats clearly shows the phenomenon: these rats develop arthritis and colitis in the presence of microbiota but are protected in a germ-free environment [77]. Thus, microbiota can be a decisive environmental factor, and new studies suggest possible causal links between microbiota and CIDs [78].

### Pleiotropy theory of CIDs

George C. Williams, an evolutionary biologist studying aging [79], stated in 1957:

It is necessary to postulate genes that have opposite effects on fitness at different ages, or, more accurately, in different somatic environments.

This theory is now widely accepted in aging research and elsewhere, and it is called ‘antagonistic pleiotropy theory’. The word pleiotropy comes from the Greek pleio, meaning ‘many’, and trepein, meaning ‘influencing’. Pleiotropic means that genes can influence different phenotypic traits at different life history points. We propose that the same theory applies to the situation in CIDs, with genes being adaptive at an early age, but maladaptive at older age (another somatic environment), leading to an overall increase in Darwinian fitness, even though causing disease and suffering at older age (Table 3).

**Table 3.** Examples of antagonistic pleiotropy for genes that increase risk or severity of chronic inflammatory diseases [80]

Genes	Chronic inflammatory disease	Pleiotropic meaning outside of chronic inflammatory diseases (with selection advantage)	Refs.
HLA DR4 (DRB1*04)	Rheumatoid arthritis and other autoimmune diseases	Decrease of risk of Dengue hemorrhagic fever (defense against infectious agents)	[81]
Fc-gamma receptor IIIA, 158 valine/valine	Rheumatoid arthritis and other autoimmune diseases	Decrease of poliomyelitis infection due to strong natural killer cell activity (defense against infectious agents)	[82]
HLA B27	Ankylosing spondylitis and other axial forms of spondyloarthritis	Decrease of viral infection (defense against infectious agents)	[83, 84]
PTPN22 1858 C > T*	Many autoimmune diseases	Improved storage of energy-rich fuels (higher body mass index, higher waist-to-hip ratio in women)	[85]
CTLA4 49 A > G	Many autoimmune diseases	Better defense against hepatitis B virus and helicobacter pylori (defense against infectious agents)	[86, 87]
NOD2/CARD15	Crohn’s disease	Hypertension (activation of the sympathetic nervous system and, thus, the fight-and-flight response)	[88]

It can be expected that all genes associated with CIDs were positively selected because they confer fitness benefits by improving reproduction, stronger skeletal muscles, better storage of energy-rich fuels (in fat tissue), or better fight-and-flight responses (stronger activation of the sympathetic nervous system and HPA axis). However, a gene or allele increasing reproductive success in early adult life can play a deleterious role in an elderly person suffering from a CID, and still overall increase Darwinian fitness. For example, a gene may confer an excellent immune response against infectious agents because it leads to a stronger activity of immune cells or better bacteria/virus recognition. This gene is advantageous in younger ages because the gene carrier has less childhood infections and, thus, a higher chance to survive until reproductive age. However, the immune system of the gene carrier might be over-activated later in life, leading to the development of a CID. However, this cost at late age (often in post-reproductive life history stages) would be much lower than the benefit of increased chance of survival until reproduction.

**Figure 1.**
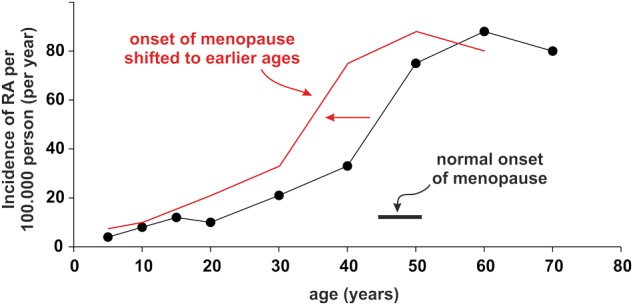
Incidence rate of rheumatoid arthritis (RA). The black line indicates the normal situation with a menopause starting at 45 years of age. The red line demonstrates a fictitious situation with accelerated menopause years before

Let us make another example from the endocrine world. It is widely accepted that the incidence rate of rheumatoid arthritis in women largely increases after menopause [89] (Fig. 1). Women with an early onset of menorrhoea, with shorter cycle lengths and higher numbers of ovulatory cycles have an increased risk of early menopause [90, 91]. In order to understand this phenomenon, one has to recall that ovaries are equipped with a limited number of oocytes, and, with every ovulation, oocytes are released and the remaining number decreases gradually. Under consideration of an early onset of menorrhoea and shorter ovulatory cycles, more oocytes will be released until age 40.

The occurrence of menorrhoea and premature adrenarche has a strong genetic component [92]. Under some environmental conditions, the carrier of this trait should reproduce earlier and more often, thereby, increasing reproductive success. However, this trait also causes earlier menopause, increasing the risk to develop the CID rheumatoid arthritis [59, 89], but the cost of developing this CID might be low compared to the fitness benefits of early and frequent reproduction. Thus, a gene conferring an early onset of menorrhoea that might be favorable for sexual function and reproduction early in life, would lead to early menopause and an earlier onset of rheumatoid arthritis (red line in Fig. 1) [59]. In addition, RA patients with an earlier onset of menopause have a more severe disease with more pain and positive rheumatoid factors [93].

The link between the HLA system, infection, and sets of secreted cytokines was nicely summarized by Chella David’s group of the Mayo Clinic [43]. This investigation demonstrated that cytokine sets are related to different types of infectious immune stimuli. A more clinical example is given in a Mexican population. It was demonstrated that the genetic risk factor HLA-DR4 (DRB1*04), known to be positively associated with rheumatoid arthritis and other CIDs, is highly negatively associated with the risk of dengue hemorrhagic fever [81]. HLA-DR4 (DRB1*04) homozygous individuals were 11.6 times less likely to develop dengue hemorrhagic fever in comparison to DR4(DRB1*04) negative persons, demonstrating that the genetic factor HLA-DR4 (DRB1*04) is protective against this infectious disease [81]. Since we are all descendants of African *Homo sapiens*, dengue was relevant to the entire human population some 100 000 years ago when humans lived in warmer climates. Thus, it seems that HLA-DR4 (DRB1*04) has been evolutionarily positively selected to fight off dengue hemorrhagic fever and other microbes in early life but later in old age supports the development of a CID. This leads to life-time reproductive success by its action in early life, even though imposing costs by increasing the risk to develop CIDs at late age.

Another example is the Fc-gamma receptor IIIA, which is responsible for the recognition of immunoglobulin G leading to immune cell activation. The recognition of immunoglobulin is important to detect bacteria and viruses, which are bound to immunoglobulin G (opsonization and phagocytosis). After binding to the cell surface Fc-gamma receptor IIIA, immunoglobulin G together with bacteria or viruses are taken up, and the cell gets stimulated to destroy the microbe. However, one form of the Fc-gamma receptor IIIA, with a genetic polymorphism in position 158, being homozygous for the amino acid valine (158 valine/valine), has a higher binding affinity for immunoglobulin G when compared to other Fc-gamma receptors [94], and this is associated with a higher risk to develop rheumatoid arthritis [95]. Importantly, the 158 valine/valine variant seems to protect against poliomyelitis [82], which indicates that this receptor variant confers protection against viral disease. Again, it seems that the genotype 158 valine/valine of the Fc-gamma receptor IIIA has been evolutionarily positively selected to fight off infectious agents by increasing microbe clearance. This trait is most advantageous in early ages (infants and young adults) by increasing the chances to reproduce, but the same surface molecule assists the development of a CID in older ages.

Pleiotropy occurs when a single gene influences multiple phenotypic traits. Such a gene can be largely advantageous for the carrier in earlier years in reproductive time, but it can also be highly disadvantageous in later ages in another somatic environment such as aging [79] or CIDs. These considerations might open our eyes when we study genetic risk factors in patients with CIDs. It also explains why a risky gene polymorphism is most probably not specific for a given CID, because the gene was evolutionarily positively selected for a different function.

## IMMUNE RESPONSE, ENERGY CONSUMPTION VERSUS PROTECTION and CIDs

In a previous review, it was argued that a highly activated immune/repair system should not be switched on for a long time because this would be very energy-consuming [14]. Additionally, a highly activated immune system is accompanied by sickness behavior and anorexia, which prevents adequate food intake and necessitates life on stored reserves (inflammation-induced anorexia). Under systemic inflammatory conditions, breaking down all reserves takes 19–43 days [14]. An energy trade-off between immune system and other bodily systems explains this time-point, and this time-point also explains when inflammation becomes maladaptive and chronic (like in CIDs) [14].

A highly activated immune/repair system can need huge amounts of energy. For example, the energy consumption in the case of extensive burn wounds (up to 20 000 kJ/day [4777 kcal/day]) [14] is approximately the same as during military jungle training (also 20 000 kJ/day; ref. [96]), and more than during military arctic training (∼18 000 kJ/day; ref. [97]). Although burn wounds represent the extreme end of the spectrum, it demonstrates the high energy consumption of the immune system (another number on energy expenditure of the immune system is 15 000 kJ/day in sepsis). Energy is a limited resource for all vertebrates, a key determinant of survival and reproduction.

Energy consumption and energy storage are some of the most critical determinants in evolution [98]. If alterations of homeostasis lead to the selection of physiological mechanisms characterized by very high energy consumption, then the situation cannot be chronic—it must be acute. Such situations lead to allostatic overload type I, which means that energy consumption is higher than energy intake, which occurs during emergency life history stages [98]. Since time to total consumption of stored energy is ∼19–43 days [14], an acute energy-consuming change of homeostasis must be started and terminated within this time frame, as otherwise permanent damage and finally death occur.

A very good example for this time window is the germinal center reaction of B cell expansion and contraction that happens within ∼21–28 days [99]. Most acute disease states are terminated within this time frame such as infectious diseases, wound healing, and wound repair, but also strong mental activation in catastrophic stressful situations must be terminated because they are highly energy-consuming [100]. During evolution, respective homeostatic networks of supersystems were positively selected for short-lived acute energy-consuming responses but not for long-standing polygenic CIDs or chronic mental illness. These chronic situations would have generated a strong negative selection pressure if they occurred during early life and during the reproductive life history stage.

In contrast, if mutations were helpful to protect energy reserves, they were positively selected during evolution. This is true for memory responses because immediate reaction of an educated system can spare energy reserves. This is exemplified by the immune memory that leads to shorter, more effective and, finally, less energy-consuming reactions towards microbes. Importantly, acquisition of immune memory during the primary contact must fit into the above specified time frame of 19–43 days (and this happens as exemplified by the germinal center reaction in secondary lymphoid organs, ref. [99]). In this context, immunological tolerance versus harmless foreign antigens (e.g. of microbes on the skin) or harmless autoantigens is a memory function that spares energy reserves.

Another example of positively selected gene variants are genes for food intake and fat storage, both of which are important in determining above-mentioned total consumption time. Indeed, for a female *Australopithecus afarensis* it has been estimated that the consumption time was ∼19 days, while in a modern female *Homo sapiens* it is 43 days [14]. In human evolution, fat storage has been markedly increased over the last 6 million years [101]. Not surprisingly, the latest metaanalysis of genome-wide association studies of obesity and the metabolic syndrome found polymorphisms in genes relevant for food intake such as *FTO* (fat mass and obesity related), *MC4R* (melanocortin receptor type 4), *POMC* (proopiomelanocortin, the precursor of melanocortin) and genes relevant for fat storage such as the insulin-stimulating *GIPR* (gastric inhibitory polypeptide receptor) [102].

We explained these common signs and symptoms as a trade-off between energy allocation to the immune system *versus* distribution to the rest of the body. The immune system is selfish [103], because a fast and strong response is needed during infection to enable survival to future reproduction [104]. For example, in*Drosophila melanogaster*, which shares a common ancestor with us 530 million years ago, it was demonstrated that the immune system is selfish in the form that it requires a lot of energy which is then not available for other systems [104]. The authors described systemic changes in energy metabolism during an infectious immune challenge by extracellular adenosine. They found that extracellular adenosine, released from immune cells, induces a metabolic switch characterized by the suppression of nutrient storage and developmental growth in favor of the immune defense. This metabolic switch—a tradeoff between development and defense—is crucial for the resistance to infection. In Drosophila larvae lacking adenosine signaling, development is not suppressed and the resistance dramatically drops [104].

In sum, genes relevant for functioning of the supersystems are positively selected to protect energy stores under well-defined conditions (Table 4). Immune mechanisms were positively selected for either acute, highly energy-consuming responses terminated within 3–8 weeks or long-standing, energy-protective responses.

**Table 4.** Positively selected immune mechanisms under defined conditions of A) acute, highly energy-consuming responses terminated within 3–8 weeks and B) long-standing, energy-protective responses

Positively selected for acute, highly energy-consuming situations	Positively selected to protect energy stores
Immune response due to infection	Tolerogenic immune reactions
Immune response to foreign bodies	Control of inner and outer body surfaces
Clonal expansion and apoptosis	Memory of the immune system
Wound healing, burn wounds	Replacement of cells and tissue (physio logical regeneration and degeneration)
Implantation of stems cells into injured tissue	Implantation of a blastocyst into the uterine epithelium
Specific immunoglobulin production and affinity maturation	Immune phenomena facilitating semiallogenic pregnancy
High production rate of cytokines and chemokines	Allergic reactions (preformed response to clear or block threats on body surfaces)
Increased rate of phagocytosis	
Immune stimulated neoangiogenesis and wound healing	

The list is not complete.

## MICROBIOTA, ENERGY REGULATION AND CIDs

Above, we mentioned that microbiota play an important role in HLA-B27 transgenic rats, which only develop CIDs when exposed to microbiota [77, 105]. Some discussed the influence of microbiota on development of CIDs simply as an unfortunate trigger of the innate and adpative immune systems leading to autoimmunity. However, microbiota also play an enormous role in metabolism and, thus, in energy regulation of the body [106, 107].

In experimental animals, there exist causal links between gut microbiota and obesity, which demonstrates an influence on energy regulation [107]. Conventionally raised mice develop diet-induced obesity, while germ-free animals do not [106]. Some bacterial divisions seem to play an important role for obesity [107, 108]. The obesity-triggering microbiome has an increased capacity to harvest energy from the diet [108], but it is also linked to more inflammation in the gut and liver [109, 110].

One may speculate that some bacterial divisions are important in establishing a more proinflammatory milieu supported by a higher degree of energy stores (obesity) to overcome acute infectious illness (free fatty acids as energy-rich fuels). Thus, the change in the microbiome can be positive for short-lived inflammatory diseases. However, the chronic change of the microbiome towards these proinflammatory bacterial divisions might be accompanied by chronic inflammatory illness. One has to keep in mind that bacterial divisions are also relevant for immunoregulatory function. Thus, it needs to be determined which groups play a causal role in the course of a CID.

## The ‘NON-SPECIFICITY’ of SIGNS AND SYMPTOMS IN CIDs

In medicine, usually, the signs and symptoms in CIDs are thought to be an accident of the disease. The ultimate cause of CIDs and typical disease sequelae is not known. Symptomatology appears to be baffling and there is no common denominator to explain often common systemic disease sequelae in CIDs. In addition, physicians still believe that many symptoms are specific for one or the other CID, which most often is not the case.

At this point we recall that CIDs-related gene polymorphisms were not specifically selected for a given CID. In contrast, because of strong negative selection for these gene variants, natural selection would have outselected these genes. Thus, evolutionary medicine asks why these genes have not been outselected. In CIDs, which typically occur in a post-reproductive life history stage, uniform pathways and networks were positively selected as adaptive physiological mechanisms to deal with infections and wound healing in life history stages before or during reproduction (Table 4). We call this uniformity a non-specificity of CIDs.

This non-specificity is obvious in clinical medicine because symptoms in various infectious diseases including CIDs are surprisingly similar. Think of the erythrocyte sedimentation rate, which most often climbs independent of the type of acute inflammation or CID. Or think of fatigue, which often accompanies acute inflammation or CIDs. Many more common signs and symptoms have been recently summarized (Table 5) [13, 14, 103, 111–113].

**Table 5.** Common signs and symptoms in chronic inflammatory systemic diseases

Overt symptoms	Change
Amenorrhea	Increases
Avolition	Increases
Body temperature and sweating	Increases
Bone loss	Increases
Cachexia, cachectic obesity	Increases
Circadian rhythms of symptoms	Become apparent
Coagulation system	Activated
Disposition to pain (skeletal muscle, joints, other)	Increases
Erythrocyte sedimentation rate	Increases
Fatigue	Increases
Food intake, appetite (finally body weight), malnutrition	Decreases
Headache	Increases
Heart rate, sympathetic nervous tone	Increases
Hemoglobin per erythrocyte, inflammation-related anemia	Decreases
Hypertension and volume expansion/water retention	More often
Insulin resistance	Increases
Interleukin-6 serum levels (one example of a cytokine in the blood)	Increases
Libido, erectile dysfunction (loss of activity of the HPG axis)	Decreases
Numbness	Increases
Parasympathetic tone	Decreases
Physical activity	Decreases
Proinflammatory high density lipoproteins (HDL), dyslipidemia	Increases
Protein in the urine	Increases
Serum albumin	Decreases
Sleeping problems	Increases
Stress negatively influences inflammation	Becomes apparent
Symptoms of depression	Increases
Vertigo	Increases
Weakness	Increases

This list is not complete. HPG axis, hypothalamic-pituitary-gonadal axis.

We explained these common signs and symptoms as a trade-off between energy allocation to the active immune system *versus* the rest of the body. While the presence of these phenomena is useful for short-lived inflammatory episodes in order to optimise recovery (it is not accident), long-term use in CIDs of the same programs is maladaptive.

## CONCLUSIONS

**Figure 2.**
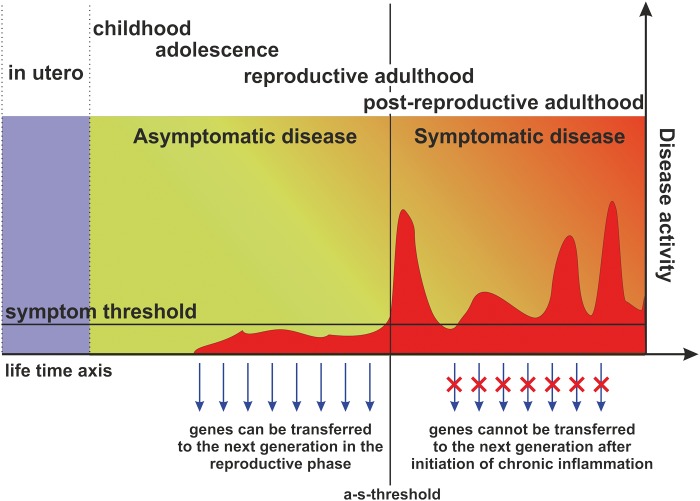
There is a critical difference between typical inflammation and chronic inflammatory systemic diseases, which separates the **a**symptomatic phase from the **s**ymptomatic phase of a chronic inflammatory systemic disease, the asymptomatic-symptomatic-threshold (a-s-threshold). Importantly from an evolutionary point of view, reproduction is only impeded during the symptomatic, but not during the asymptomatic phase. Genes enabling an adaptive inflammatory response, allowing the organism to overcome an infection, will thus increase survival probability and the potential for future reproduction and as such evolutionary fitness in young age. After initiation of chronic systemic inflammation, however, reproduction is inhibited, and this for prolonged periods, often for years and until death, causing fitness costs. However, as these costs typically occur only at the end of the reproductive life-history stage or in post-reproductive age, these fitness costs are lower than the fitness benefits in early life leading to an overall increase in Darwinian fitness

CIDs lead to great and long-term suffering, imposing stress on people and high costs on society. During evolution, respective homeostatic networks of supersystems involved in CIDs were positively selected for short-lived acute energy-consuming responses but not for long-standing polygenic CIDs (Fig. 2). Several genetic factors of these supersystems that are favoring the development of CIDs have been identified. These factors typically increase fitness in young pre-reproductive and reproductive age, and resulting fitness benefits are expected to be higher than the fitness costs in post-reproductive age. Thus, life-time reproductive success will be increased, explaining why factors favoring CIDs have not been outselected but—under specific environmental conditions—have even been positively selected. Different CIDs result from uniform pathways and networks, which has been called the non-specificity of CIDs. For clinicians and the pharmaceutical industry to understand maladaptive responses in CIDs, they first need to comprehend adaptive functions in early life.

## FUNDING

For this work, the authors were funded by their institutions.

**Conflict of interest**: None declared.
